# A Case of Severe Tricuspid Regurgitation Related to Traumatic Papillary Muscle Rupture

**DOI:** 10.1155/2020/8505894

**Published:** 2020-03-31

**Authors:** Ruchika Meel, Bongane Ngutshane, Ricardo Gonçalves, Shungu Mogaladi

**Affiliations:** ^1^Department of Internal Medicine, Division of Cardiology, Chris Hani Baragwanath Hospital, University of the Witwatersrand, Johannesburg, South Africa; ^2^Department of General Surgery, Division of Cardio-Thoracic Surgery, Charlotte Maxeke Hospital, University of the Witwatersrand, Johannesburg, South Africa; ^3^Olivedale Hospital, Johannesburg, South Africa

## Abstract

A 25-year-old male presented after a motor vehicle accident with tricuspid valve (TV) regurgitation, due to a flail TV secondary to papillary muscle rupture. We highlight the importance of three-dimensional echocardiographic imaging of the tricuspid valve and its utility in aiding a successful surgical repair.

## 1. Introduction

The majority of injuries to the tricuspid valve (TV) apparatus are related to blunt chest trauma [1]. Motor vehicle accidents (MVA) are a common cause of blunt trauma to the heart. It is unusual for the TV to be involved in isolation [2–4]. The aortic valve is most commonly involved, followed by the mitral valve and finally the TV. The postulated mechanism of injury to the TV in these cases tends to be a rapid deceleration force combined with an increase in intracardiac right chamber pressures [5]. In a vast number of cases of MVA, cardiac injury tends to be overlooked due to other overt injuries [6]. Injury to the TV can be silent depending on the severity of structural damage [7]. Therefore, some authors have advocated routine and timeous use of echocardiography in patients sustaining blunt trauma to the chest [6]. The advent of newer echocardiographic techniques such as three-dimensional (3D) imaging acts as a supplementary tool in characterising the precise location of injury to the TV [8].

## 2. Case Presentation

A 25-year-old healthy male presented to a peripheral hospital in Johannesburg, South Africa, after an MVA. He was an unrestrained passenger who hit the car dashboard after sudden deceleration from high speed. He sustained multiple injuries including bilateral hemopneumothoraxes. An admission transthoracic echocardiogram (TTE) revealed severe TR of unclear mechanism.

**Figure 1:**
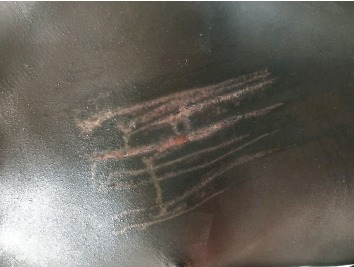
Abrasion marks on the anterior chest wall secondary to blunt chest trauma.

**Figure 2:**
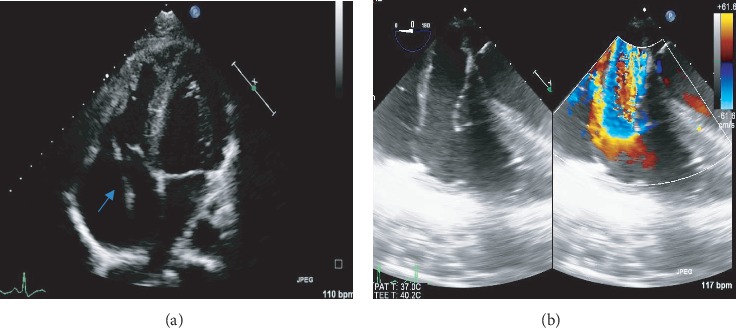
(a) Transthoracic apical 4-chamber echocardiographic view showing flail anterior tricuspid valve leaflet (arrow) and (b) transoesophageal right ventricle long axis views depicting severe tricuspid valve regurgitation.

**Figure 3:**
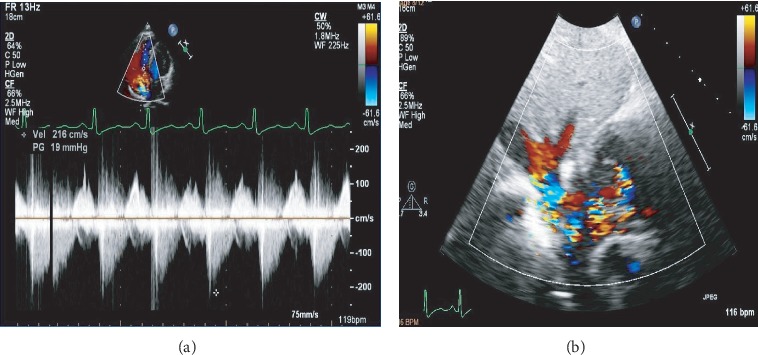
(a) Continuous wave Doppler through the tricuspid valve in 4-chamber view showing an early peaking triangular tricuspid regurgitation velocity. (b) A subcostal view showing hepatic vein systolic flow reversal due to severe tricuspid valve regurgitation on colour flow imaging.

**Figure 4:**
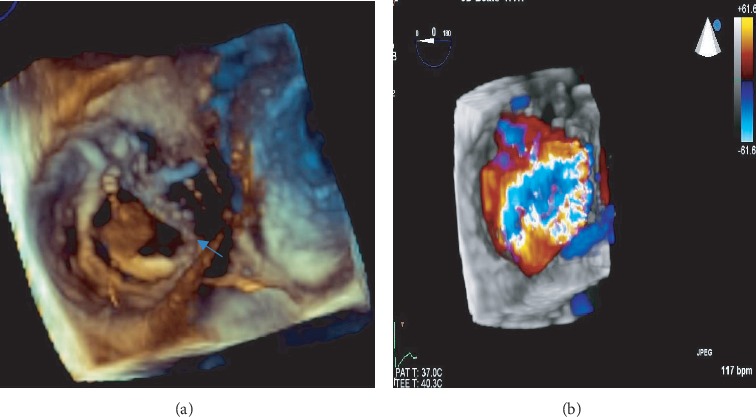
(a) Three-dimensional view of the tricuspid valve from the right atrial aspect showing flail anterior tricuspid valve leaflet and ruptured papillary muscle (arrow) and (b) three-dimensional colour imaging showing severe tricuspid regurgitation into the right atrium.

**Figure 5:**
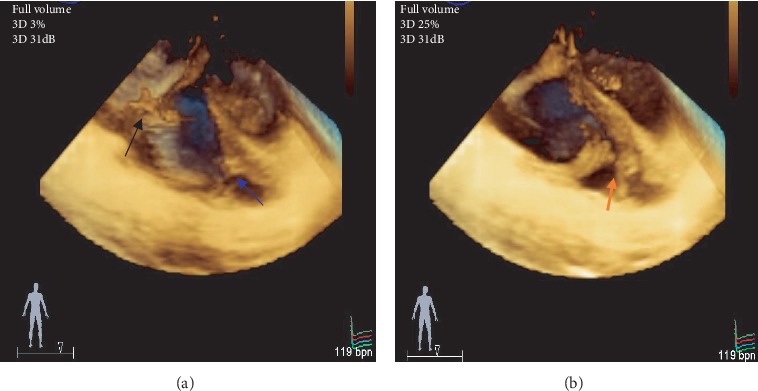
Three-dimensional transoesophageal views of the right ventricle showing the attachment point of the anterior papillary muscle ((a) blue and (b) orange arrows) and flail anterior tricuspid leaflet with ruptured papillary muscle ((a) black arrow).

He was referred 2 weeks after the index admission to a tertiary hospital. On arrival, he was asymptomatic and haemodynamically stable. He had abrasion marks on the chest (Figure 1). Cardiovascular examination revealed prominent internal jugular vein c-v waves and a soft grade 2/6 pansystolic murmur at the left lower parasternal border. He was not in heart failure. Sinus tachycardia and an early repolarisation pattern were noted on the 12-lead electrocardiogram. He had normal laboratory blood parameters. On TTE, a moderately enlarged right ventricle (RV) and right atrium, with preserved RV systolic function, was noted. There was a flail TV leaflet and an oscillating mass in the right atrium (Figure 2(a)). Avulsion of the anterior papillary muscle from the RV wall was suspected. The colour flow Doppler revealed severe TR with an early peaking triangular jet velocity of 2.16 m/sec (Figures 2(b) and 3(a)). There was systolic flow reversal in the hepatic veins (Figure 3(b)). A 3D transoesophageal echocardiogram (TEE) was performed which confirmed a flail anterior TV leaflet due to rupture of the anterior papillary muscle (Figures 4(a), 5(a), and 5(b)). On 3D colour flow, the TR was severe, with the jet filling greater than 50% of the right atrium (Figure 4(b)).

**Figure 6:**
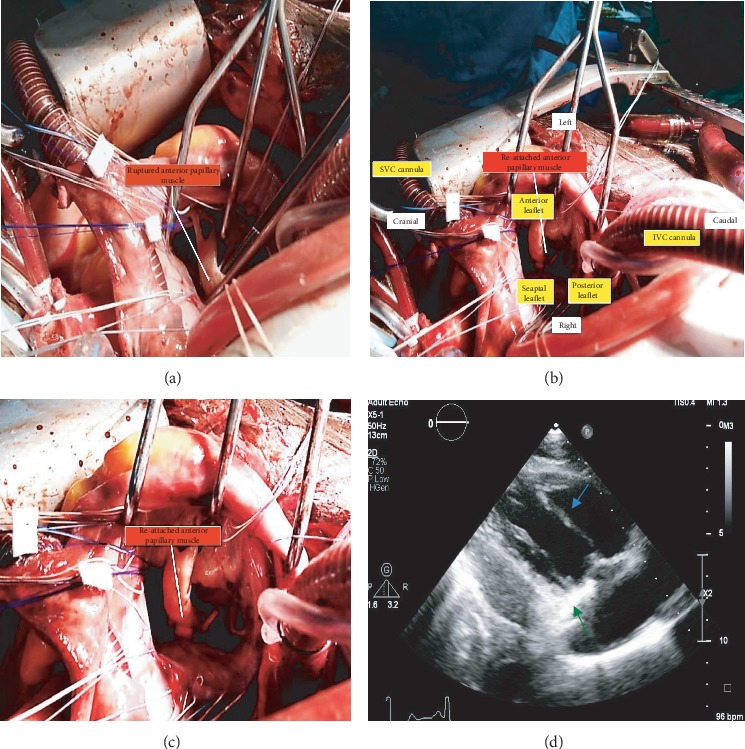
(a–c) Intraoperative images of ruptured anterior papillary muscle and its repair and (d) transthoracic postoperative right ventricular inflow view showing the repaired anterior papillary muscle (blue arrow) and annuloplasty ring (orange arrow).

The patient underwent a successful surgical repair of the TV (Figure 6). A flail anterior TV leaflet due to anterior papillary muscle rupture was identified with a residual papillary muscle stump on the RV. The anterior papillary muscle was reattached to the stump with pledgeted 4-0 polypropylene sutures. Additionally, a modified De Vega annuloplasty was done. Finally, the valve competency was tested by saline injection into the RV and confirmed by TEE after coming off cardiopulmonary bypass. Trivial TR was noted. The patient had an uneventful postoperative course.

## 3. Discussion

Our case brings attention to the following pertinent aspects related to traumatic tricuspid valve insufficiency: (1) underreporting of TV injury in Africa and the importance of meticulous screening for TV injury in patients with history of blunt chest trauma; (2) the value of three-dimensional imaging of the TV prior to surgical referral; and (3) early referral for surgery of patients with severe TR due to TV injury.

Motor vehicle accidents are an important cause of TV injury [9, 10]. South Africa has one of the highest motor vehicle accident-related fatalities [11, 12]. Yet, there is a paucity of data regarding TV trauma related to MVA. This is possibly due to TV injuries being missed in a polytrauma patient, lack of resources in terms of imaging in low- and middle-income countries, or underutilisation of existing imaging modalities due to a lack of expertise. Additionally, the true prevalence of TV regurgitation due to trauma is underreported as patients experience minimal or no symptoms for a prolonged period of time [13]. TTE is a useful bedside tool for assessing cardiac structure and function in a trauma patient [14]. It is freely available at most institutions, noninvasive, inexpensive, and radiation- and contrast-free [15]. It has been validated as a useful tool for right heart, TV, and subvalvular apparatus assessment. Transoesophageal echocardiography (TEE) can be utilised in cases where TTE provides insufficient information for decision making^14^. However, TEE is not widely available and needs specialized expertise.

Recently, more attention has been focused on 3-dimensional imaging of the TV [16]. It allows “enface” views of the valve and thus easy discrimination of the 3 TV leaflets. Accurate anatomical information aided in planning this TV repair by identifying the exact site anterior papillary muscle rupture. The literature suggests a lower success rate for surgical repair when the mechanism for TV regurgitation is not papillary muscle rupture [13]. In the current era of 3D imaging, successful TV repair for TR due to papillary muscle rupture has been reported [17].

A surgeon with sufficient skill and experience in repairing tricuspid valves is crucial, and all imaging is complementary to direct anatomical inspection. Early referral of patients with severe TR is advised to prevent right ventricular dysfunction from chronic volume overload [13]. It has been noted that if the operation is delayed, valve repair becomes more challenging due to the development of fibrosis of the valve and subvalvular apparatus [18]. For these reasons, early surgical referral was preferred and led to a successful outcome. Follow-up of the patient is important as TR may recur.

In conclusion, we have presented a case of successful TV repair with the aid of 3D echocardiographic imaging from Africa. We hope that this case will stimulate readers to actively search for and report on TV-related injury in a patient with a history of chest trauma.
